# Activated Carbon and Syntrophy Accelerate the Corrosion of Stainless Steel Under Strict Anaerobic Conditions by *Methanosarcina barkeri*

**DOI:** 10.3390/microorganisms13061278

**Published:** 2025-05-30

**Authors:** Chunyu Zhou, Shiqi Huang, Haoyong Li, He Dong, Haowen Zhang, Wenwen Chen, Yan Dang

**Affiliations:** Beijing Key Laboratory for Source Control Technology of Water Pollution, Engineering Research Center for Water Pollution Source Control and Eco-Remediation, College of Environmental Science and Engineering, Beijing Forestry University, Beijing 100083, China; zhouchunyu1111@126.com (C.Z.); huangshiqi07142022@163.com (S.H.); haoyongli@bjfu.edu.cn (H.L.); hedong1997@bjfu.edu.cn (H.D.); zhanghw1998@bjfu.edu.cn (H.Z.); chenww0107@163.com (W.C.)

**Keywords:** stainless steel, corrosion, extracellular electron transfer, granular activated carbon, *Methanosarcina*, *Geobacter*

## Abstract

Previous studies have demonstrated that some methanogens can directly accept electrons from Fe(0), leading to metal corrosion under strict anaerobic conditions. However, there are few reports on the research of anaerobic iron corrosion by some substances that coexist with methanogens, such as syntrophic bacteria and activated carbon, which is widely distributed in environments. Therefore, in this study, a corrosion system consisting of *Methanosarcina barkeri*, stainless steel, and granular activated carbon (GAC), as well as a corrosion system with *Geobacter metallireducens*, was constructed. The aim was to explore the mechanism of stainless steel corrosion under the metabolic action of *M. barkeri*. It was found that the GAC and *G. metallireducens* can accelerate the extracellular electron transfer between *M. barkeri* and stainless steel, thereby accelerating corrosion, and this intensification mechanism may be related to the *mtmC*, *mtbC*, and *fwdC* genes. By understanding these mechanisms, not only can a theoretical basis be provided for the protection against metal corrosion, but it can also promote environmental protection and safe production.

## 1. Introduction

Wear and corrosion resistance are fundamental requirements for materials subjected to aggressive environments. Stainless steels are notable due to their combination of excellent mechanical properties and superior corrosion resistance [1]. They are widely employed in construction, food industry, chemical industry, medical equipment, aerospace, energy, and other fields. The damaging effect of corrosion is supported by estimates of the damage caused, which account for approximately 10–15% of the global direct metal production losses. However, the actual damages are far greater because to the direct losses must be added the indirect losses determined by loss of production, loss of capacity, and contamination of products [2]. In recent years, with the increasing complexity of the industrial environment, the corrosion resistance of traditional materials is facing greater challenges. Furthermore, the anaerobic corrosion of iron structures is expensive to repair.

It has been known for over 100 years that the presence of anaerobic respiratory microorganisms can accelerate iron corrosion [3]. Microorganisms can corrode either through metabolites or by forming biofilms on metal surfaces. The microbial metabolic activities in the biofilms will change the pH value of the local environment and thus affect the corrosion efficiency [4]. Thus, it is indisputable that the microbial corrosion or bio-corrosion plays a predominant role, with considerable economic losses. Despite many microbial species being described as corrosive, certain groups, such as sulfate-reducing bacteria [5] and nitrate-reducing bacteria, acetogenic bacteria, and methanogenic archaea, are the most commonly implicated [6]. Tang [3] has reported that *Geobacter sulfurreducens* strain ACL can extract electrons from Fe(0) by hydrogenase and C-type cytochrome on the outer surface. Previous studies have inferred that methanogenic microorganisms accelerate the corrosion of iron-containing metals. Holmes [7] report that *Methanosarcina acetivorans*, which also belong to the genus *Methanosarcina*, obtain electrons from metals to produce methane, which enhances the possibility of *M. barkeri* corroding stainless steel.

*Methanosarcina barkeri* is also a methanogenic archaea widely distributed in the environment, a very versatile methanogen using hydrogenotrophic, methylotrophic, and acetoclastic methanogenesis [8]. In recent years, *M. barkeri* has gradually become one of the hot spots of research due to its potential in biodegradation and energy recovery. However, *M. barkeri* not only plays an important role in anaerobic digestion wastewater treatment [9] and biogas upgrading [10] but also takes Fe(0) as a potential electron donor in some extreme environments [11], causing metal corrosion, especially in anaerobic environments. In these environments, microorganisms may affect the stability of metal surface films by producing acids, producing gases (such as methane, hydrogen), and excreting other metabolic byproducts. It has been reported, for example, that planktonic cells of *M. fulgenii* can form biofilms on iron films and improve the biological corrosion activity during anaerobic iron corrosion, thus accelerating the corrosion process of metals [12]. In addition, as an anaerobic microorganism, the growth and metabolic activities of *M. barkeri* often reduce the oxygen concentration in the local environment, so that the microorganisms directly absorb electrons from metal iron through extracellular electron transfer to increase the electrochemical reaction rate of corrosion [13]. In recent years, it has been found that in the environment where *M. barkeri* exists, the corrosion rate of stainless steel and other metal materials increases significantly [14].

With the aggravation of environmental pollution, activated carbon has been applied to pollutant treatment in an increasing number of industries, including the treatment of dairy wastewater [15], the removal of pesticides in the atmosphere [16], and the removal of pollutants in surface water [17]. This has led to the widespread presence of carbon particles in the ecological environment. Granular activated carbon (GAC) is a highly porous carbon material with a wide surface area and excellent adsorption properties. These properties make it an effective material for a variety of applications, including water treatment [18] and electrochemical reactions [19]. In anaerobic digestion of wastewater, GAC is often used to promote electron transfer and thus increase the methane production rate due to its excellent electrical conductivity [20]. This indicates that the conductive properties of GAC may promote electron transfer between microbial cells and metal surfaces, affecting the corrosion of materials such as stainless steel caused by microorganisms. A study has shown that *Geobacter* and *Methanosarcina* often coexist in a variety of anaerobic environments, including sediments, hot springs, animal gastrointestinal tracts, and rice fields [21,22,23]. *Geobacter metallireducens* can cooperate with *Methanosarcina barkeri* to enhance the metabolic activities of the two microorganisms and promote the production of metabolites [24]. Thus, it is hypothesized that the interaction between *G. metallireducens* and *M. barkeri* can also accelerate the corrosion of stainless steel.

These phenomena have attracted extensive attention from researchers. Previous studies have demonstrated that certain microorganisms capable of extracellular electron transfer (*G. metallireducens* and *M. acetivorans*) can corrode stainless steel [7,25]. Meanwhile, GAC and syntrophy (*G. metallireducens*) in co-culture systems have been shown to accelerate extracellular electron transfer [24,26]. However, due to the slow corrosion progression and long experimental duration, few studies on *M. barkeri* mediated corrosion have been reported, and the effects of GAC and syntrophy on corrosion remain unclear. Therefore, it is important to study some substances that coexist with methanogens, such as syntrophic bacteria and GAC. On the premise of having proven that GAC and *G. metallireducens* can promote electron transfer, this experiment hypothesizes that GAC and syntrophic bacteria (*G. metallireducens*) can accelerate the corrosion of stainless steel by *M. barkeri*. Based on previous studies on the corrosion of stainless steel by *M. acetivorans* and the corrosion of Fe⁰ by *G. sulfurreducens* [7,27], the experimental scheme of this study was designed.

## 2. Materials and Methods

### 2.1. Bacterial Strains and Growth Conditions

*Methanosarcina barkeri* and *Geobacter metallireducens*, purchased from DSMZ (Braunschweig, German), were routinely cultured in serum vials under strict anaerobic conditions using the N_2_:CO_2_ (80:20) mixed gas adopted in DSMZ’s official culture medium [24]. The vials were sealed with butyl rubber stoppers and aluminum rings. *M. barkeri* was cultured in DSM120 medium at a constant temperature of 37 °C [24]. The formulation of DSM120 medium is as follows: 0.5 mM Na_2_S, 1 mM cysteine, 0.002 g/L CaCl_2_·2H_2_O, 1 g/L NaCl, and 2 g/L NaHCO_3_. *G. metallireducens* was cultured in ferric citrate (FC) medium at a constant temperature of 30 °C. The formulation of the FC medium [28] is as follows: NaHCO_3_, 2.5 g/L; CaCl_2_·2H_2_O, 0.1g/L; KCl, 0.1 g/L; NH_4_Cl, 1.5 g/L; NaH_2_PO_4_·H_2_O, 0.6 g/L; as well as vitamin and trace element mixtures. Unless otherwise specified, acetate (6.8 g/L) was used as the carbon source. The gas phase was N_2_-CO_2_ (80:20). The co-culture of *M. barkeri* and *G. metallireducens* was carried out under anaerobic conditions at a constant temperature of 30 °C, using DSM120 medium, but instead of sodium acetate as the electron donor, ethanol was used [29].

### 2.2. Construction of the Culture System of Strains, Stainless Steel and GAC

The model of the stainless steel is 316 L, with the specifications of 10 mm × 10 mm × 2 mm, and the addition amount is 10 pieces per bottle. According to Liu’s experiment, when the granular activated carbon (GAC) with a particle size of 8–20 mesh is added at an amount of 25 g/L, the electron transfer efficiency between *G. metallireducens*/*G. sulfurreducens* co-cultures is optimal at 25 g/L [26]. Therefore, 25 g/L of GAC was selected for addition in this experiment. The stainless steel (SS) and GAC were immersed in acetone and then placed in an ultrasonic cleaner and soaked at room temperature for 30 min. The acetone was replaced with ethanol and then placed in the ultrasonic cleaner for 20 min. This step was repeated 2–3 times. After that, they were washed with clean water, air-dried, and placed in a desiccator. The surface of the SS was polished with three different grades of sandpaper (220, 600, and 1200 mesh), and each side was continuously polished for 60 s. Then it was soaked in 70% ethanol for 30 min and placed in a petri dish. The ethanol was poured out, and the petri dish was put into an ultraviolet cabinet for 30–45 min. During this process, the petri dish was turned over 2–3 times for sterilization [25].

During the construction of the culture system of *Methanosarcina*, *Geobacter*, GAC, and stainless steel, the stainless steel and GAC should first be placed in an aerated and anaerobic empty vial. After sealing, the vial is sterilized. Once it has cooled down after sterilization, the sterilized medium is injected into the vial using a syringe. In order to ensure that the environment in the anaerobic vial is under strict anaerobic conditions, the oxygen scavenger should be allowed to function fully as much as possible. The time of inoculation should be at least 2 h or more after the injection time of the oxygen scavenger. Three parallels are set for each group. *Methanosarcina* and *Geobacter* should be inoculated during the logarithmic phase of the cultivation process, and the inoculation amount is 20% of the content of the medium. All data obtained from the experiments were derived from three parallel groups and subjected to significance analysis using a *t*-test.

### 2.3. Analytical and Detection Methods

#### 2.3.1. Detection Method of Ions in Solution

The determination of Fe^2+^ in the solution adopts the ferrozine spectrophotometric method. The directions for this method are as follows: Take 1 mL of the sample to be tested, add 4 mL of 0.5 N HCl, and dissolve it for 15 min. Then, take 1 mL of the acid dissolution solution and add 4 mL of the ferrozine reagent. After color development for 15 min, measure the absorbance at 562 nm.

#### 2.3.2. Detection Method of Ions in Granular Activated Carbon

After the experiment is completed, take out all the granular activated carbon in the anaerobic vial, soak it in 50 mL (close to the volume of the experimental system) of 1 M hydrochloric acid solution for 2–3 h, and keep stirring or slightly shaking to ensure that the acid solution can fully dissolve the iron ions in the activated carbon, obtaining the iron–ion eluent of GAC. The ferrozine spectrophotometric method is used for measurement.

#### 2.3.3. Determination of the Concentration of Substances in Solution

With a syringe, draw 1 mL of the sample from the anaerobic vial and filter it (using a 0.22 μm filter), then determine the concentration of the substrate sodium acetate or ethanol. The acetic acid in the solution is determined by a liquid chromatograph (SCION LC6000, Beijing, China) [30], with a mobile phase of 5 mM H_2_SO_4_. The ethanol is determined by a gas chromatograph (Agilent, GC7890, Beijing, China).

#### 2.3.4. Testing Method for Gas Components and Contents

Use a 1 mL syringe to sample and determine the gas products generated by the stainless-steel corrosion system. The content of the gas product CH_4_ is determined by a gas chromatograph (Tian Mei, GC7900, Zhongshan, China). The testing method follows the operation instructions of the GC7900.

### 2.4. Surface Analysis Method

A scanning electron microscope (SEM) is a commonly used surface analysis method for metals. This instrument can conduct microscopic analysis of the surface morphology of materials, thereby obtaining information such as physical morphology and the morphology of microbial films. Before sample detection, pretreatment is required. The sample is gently rinsed with 0.1 M phosphate-buffered saline (pH = 7.2). After cleaning, the sample is placed in a 2.5% glutaraldehyde solution and fixed at 4 °C for 12 h. Gradient dehydration is performed with 50%, 70%, 80%, and 90% ethanol, successively [25]. Each time, it is allowed to stand for 10–15 min and then centrifuged at 3000 rpm to remove the upper layer liquid. It is dehydrated with absolute ethanol three times. After dehydration, the sample is placed in a freeze dryer and dried for 12 h and then taken out when it is ready for monitoring. The pretreated sample is pasted onto a copper platform with conductive adhesive, and a layer of carbon is plated on the sample surface. It is then observed under a scanning electron microscope. SEM–EDS point scanning is used to measure the content of each element at the required points.

### 2.5. Detection Method for Electrochemical Indicators

An electrochemical device with a single-chamber three-electrode system was constructed. The working electrode was a stainless steel electrode, the counter electrode was a graphite plate, and the reference electrode was a KCl electrode. The solution in the device was the 120 media, which is the culture medium for *M. barkeri*. At room temperature, a scanning test was carried out in the range of −1.0 V to 0.2 V at a scanning rate of 10 mV/s. After obtaining the data, a cyclic voltammogram was plotted with the potential as the abscissa and the current as the ordinate.

### 2.6. Transcriptomics

Cells were harvested from the 50 mL anaerobic *M. barkeri* corrosion systems containing GAC and *G. metallireducens*, which had been cultivated for 60 days. Stainless steel was used as the electron donor, and the cells were grown as described above. The cells were centrifuged at 8000 rpm for 20 min at 4 °C, immediately frozen in liquid nitrogen thereafter, and stored at −80 °C [31]. RNA was extracted from the samples using the e.zn.a.^®^ Soil RNAMidi Kit (Omega Bio-Tek, Norcross, GA, USA). The concentration and purity of RNA were detected by NanoDrop2000 (Thermo Fisher Scientific, Waltham, MA, USA), and the integrity of RNA was examined by 1% agarose gel electrophoresis. The RIN (RNA integrity number) value was determined using the RNA 6000 Nano Kit on an Agilent 2100 (Beijing, China). Subsequent steps, including library construction, high-throughput sequencing, and data preprocessing, were completed by Majorbio Bio-Pharm Technology Co., Ltd. (Shanghai, China). The gene expression levels were presented as TPM (transcripts per million). Differential expression analysis was performed using the DESeq2 software, which employs the negative binomial distribution model and generalized linear model (GLM) as its core statistical methods, to identify genes with significantly differential expression across sample groups. Subsequently, Gene Ontology (GO) functional enrichment analysis and KEGG (Kyoto Encyclopedia of Genes and Genomes, Kyoto, Japan) pathway enrichment analysis were carried out on these genes to explore their potential biological functions and the signaling pathways they participated in, thus comprehensively elucidating the characteristics of transcriptome changes and their biological significance.

## 3. Results and Discussion

### 3.1. GAC Can Accelerate the Corrosion of Stainless Steel by Methanosarcina barkeri

**Figure 1:**
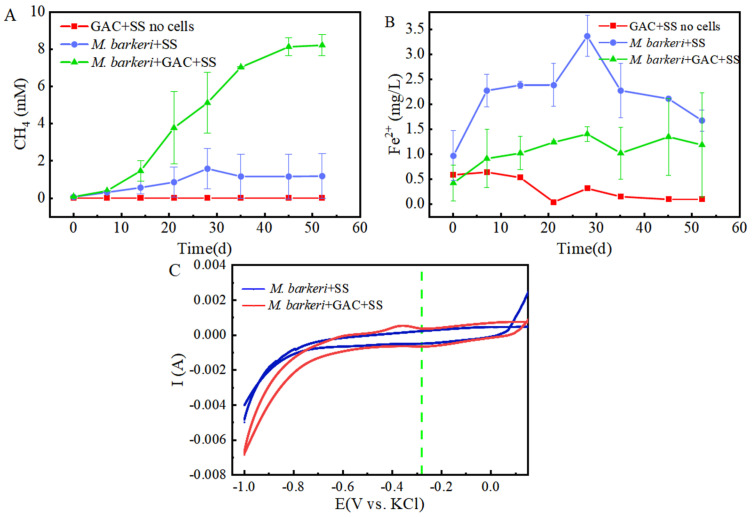
Contents of CH_4_ (**A**) and Fe(II) (**B**) changing with time in different experimental groups and the cyclic voltammogram (**C**) during the corrosion of stainless steel by *M. barkeri*.

It is known that *M. barkeri* can produce methane by obtaining electrons through direct electron transfer (DET) [32]. As a carbon-based material with a large surface area and high electrical conductivity, GAC is often added to anaerobic biogas digesters to promote methane production [33]. In this experiment, a control group consisting of GAC and stainless steel without cells was set up, with 1 mM NaAc as the electron donor to facilitate electron acquisition by *M. barkeri* from stainless steel. A system consisting of *M. barkeri*, GAC, and stainless steel was constructed to explore the enhancement effect of GAC on the corrosion of stainless steel by *M. barkeri*. Methane content, the most direct indicator of *M. barkeri* growth, was first measured, with the results shown in Figure 1A. *M. barkeri* obtained electrons from stainless steel to produce methane, and the methane content reached 1.6 ± 1.08 mM. It is worth noting that in the presence of GAC, the methane production of *M. barkeri* reached 8.22 ± 0.57 mM. When 1 mM NaAc was provided as the sole substrate, theoretically 1 mM methane could be produced (Equation (1)) [7]. However, the experimental results showed that the methane content in the two experimental groups reached 8 mM or 10 mM, indicating that stainless steel (SS) was utilized by *M. barkeri* as an additional electron donor for CO_2_ methanation. Additionally, SS does not produce hydrogen non-biologically with water; therefore, *M. barkeri* likely acquires electrons from SS via direct electron transfer (DET). This confirmed the possibility that *M. barkeri* in this growth state could attach to stainless steel and carry out electron transfer, and GAC likely accelerated electron transfer between the microbe and metal, increasing methane production.

CH_3_COONa + H_2_O → CH_4_ + CO_2_ + NaOH (1)

Meanwhile, in order to better and more intuitively evaluate the corrosion effect, we measured the Fe(II) in the culture medium, and the results are shown in Figure 1B. It can be seen that in the presence of *M. barkeri*, the concentration of Fe(II) increased, which shows that *M. barkeri* can obtain electrons from stainless steel to maintain its own metabolism. This key conclusion is highly consistent with the research finding that *Methanosarcina vacuolata*, which also belongs to type I methanogens, can obtain electrons by using stainless steel [34], further consolidating the theoretical basis for the electron transfer between microorganisms and metallic materials. It is particularly noteworthy that after introducing GAC into the anaerobic corrosion system, its methane production reached 5.14 times that of the control group. This data intuitively show the significant accelerating effect of GAC on the process of *M. barkeri* obtaining electrons from stainless steel, thereby strengthening the corrosion effect of microorganisms on stainless steel. This result coincides with the theoretical speculation put forward by Wang that GAC may enhance the generation pathway of electron shuttles and promote the intracellular and extracellular electron transfer processes [35].

**Figure 2:**
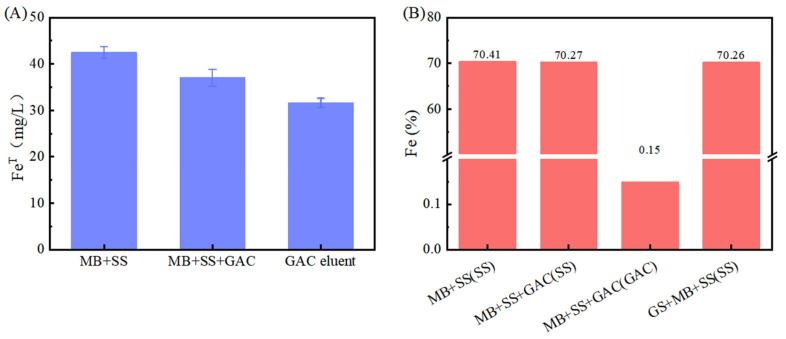
Iron content, (**A**) total iron of each liquid. Among them, “MB + SS” and “MB + SS + GAC” represent the total iron content of the bacterial liquid in the anaerobic vial. The GAC eluent is the liquid obtained by eluting the GAC in “MB + SS + GAC”; (**B**) iron element proportion near the bacterial cells obtained by SEM–EDS in each experimental group, and the scanning object is indicated in the brackets.

However, the Fe(II) concentration in the GAC-supplemented experimental group was lower than that in the group without GAC (*p* < 0.05). This is hypothesized to result from the adsorption capacity of GAC. According to the experimental data in Figure 2A, the total iron contents in the “*M. barkeri* + SS” experimental group and the “*M. barkeri* + SS + GAC” experimental group on the 52nd day were measured to be 42.49 ± 1.24 mg/L and 37.05 ± 1.83 mg/L, respectively. In addition, the total iron content in the GAC eluent was 31.60 ± 1.02 mg/L. This proves the speculation that a portion of the irons was adsorbed due to its inherent adsorption properties. Concurrently, the cumulative total iron content in the GAC group (68.65 mg/L) was significantly higher than that in the GAC-free control group (42.49 ± 1.24 mg/L) (*p* < 0.05). This result, from another aspect, proves that the addition of GAC can effectively accelerate the corrosion of stainless steel by *M. barkeri*.

To further prove the speculation, cyclic voltammetry (CV) scans were performed on the anaerobic corrosion system at the end of the experimental period, with results depicted in Figure 1C. It can be seen that the area enclosed by the scanning curve of the “*M. barkeri* + SS + GAC” group is larger than that of the “*M. barkeri* + SS” group, indicating that a larger number of *M. barkeri* adhered to the surface of the electrode, and its capacitance is greater than that of the experimental group without the addition of GAC. It is worth noting that there is a relatively obvious oxidation peak appearing at around −0.38 V in the “*M. barkeri* + SS + GAC” group, while no obvious oxidation peaks or reduction peaks are in the other one, indicating that the stainless steel underwent an oxidation reaction under the action of *M. barkeri*, losing electrons and changing into Fe(II) or Fe(III) in the solution. Meanwhile, a reduction peak appeared at around −0.28 V, indicating that a reaction occurred on the electrode in which *M. barkeri* obtained electrons and reduced CO_2_ to CH_4_.

**Figure 3:**
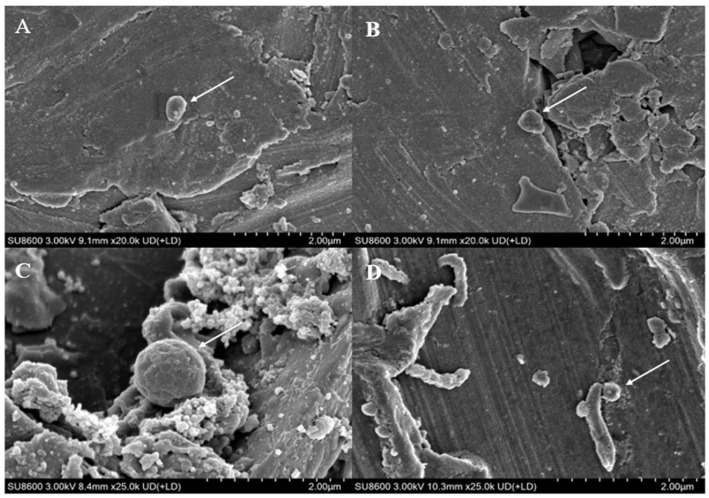
Scanning electron microscope images of different experimental groups. ((**A**): the stainless steel in the “*M. barkeri* + SS” group, and the arrow points to *M. barkeri*; (**B**): the stainless steel in the “*M. barkeri* + SS + GAC” group, and the arrow points to *M. barkeri*; (**C**): the GAC in the “*M. barkeri* + SS + GAC” group, and the arrow points to *M. barkeri*; (**D**): the stainless steel in the “*G. metallireducens* + *M. barkeri* + SS” experimental group, and the arrow points to *M. barkeri* and *G. metallireducens*).

It can be observed from the scanning electron microscope images (Figure 3) that *M. barkeri* adhered to the surfaces of the stainless steel in both groups, and the cells were in direct contact with the surfaces, without observing the accumulation between cells. Therefore, it can be reasonably assumed that there is a direct electron transfer between the metal and the microorganisms [7]. The surfaces of the stainless steel in the images show corrosion signs to varying degrees (surface roughness, local pits, or corrosion spots). For example, it can be clearly seen that the stainless steel (B) with the added GAC system has more severe corrosion cracks and corrosion pits than that (A) without the GAC system. The content of iron element near the bacterial cells is shown in Figure 2B. The atomic content diagrams and comparison tables of other experimental groups are shown in Figure S1 and Table S1. When GAC was present, the weight percent of Cr and Fe were both lower than those in the other group (*p* < 0.05), indicating that the metal oxide film on the surface of the stainless steel in the “*M. barkeri* + SS + GAC” group is more severely damaged, with more iron element loss and more serious corrosion. Meanwhile, the *M. barkeri* cells attached to the GAC and the iron weight percent of 0.15% once again prove that GAC accelerates the corrosion of stainless steel by *M. barkeri* through electron transfer.

### 3.2. Mechanism Analysis of GAC Accelerating the Corrosion of Stainless Steel by Methanosarcina barkeri

**Figure 4:**
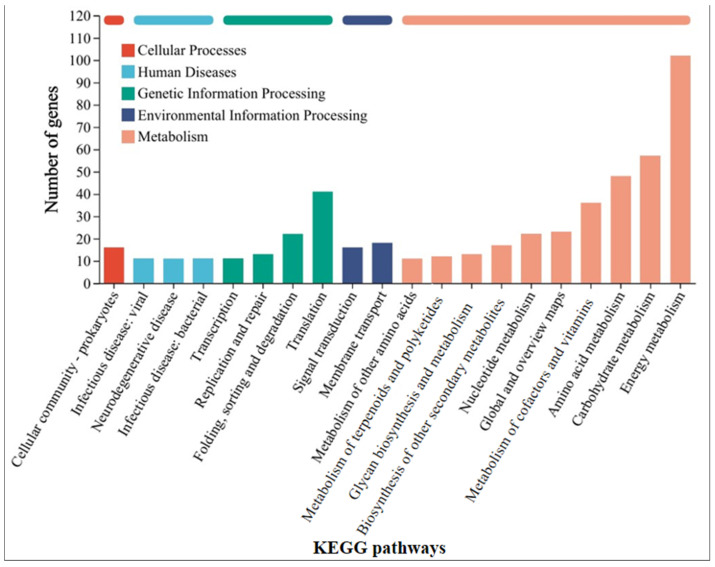
KEGG pathway classification statistics chart of differentially expressed genes between the “*M. barkeri* + SS + GAC” group and the “*M. barkeri* + SS” group.

In order to further understand the mechanism by which GAC accelerates the electron transfer between *M. barkeri* and stainless steel, the strains in the GAC-supplemented and GAC-free experimental groups were subjected to transcriptome analysis to identify differentially expressed genes. Some genes with a difference in gene expression levels of more than 1.8 times between the two experimental groups were selected, and the data shown in Table 1 were obtained. The other highly expressed genes and the specific values of each experimental group are shown in Table S2. As shown in Table 1, the gene MSBRM_2245, which is related to the editing of annexin, exhibits a relatively large expression difference. This indicates that electrons are directly transferred through the cell membrane of *M. barkeri*, which belongs to the category of direct electron transfer. In addition, the expression difference of *mtmC*, which encodes methyltransferase-like proteins, is also relatively high. The difference in the expression levels of this gene between the “*M. barkeri* + SS + GAC” group and the “*M. barkeri* + SS” group is 7.276 times (*p* = 0.034). Stephen A. Burke described how *mtmC* is an important gene for catalyzing methylation, which establishes a family of cobalamin-binding proteins involved in methylotrophic methanogenesis [36]. At the same time, there are two other genes related to methanogenesis, *cofD* and *mfnE*, with relatively large fold changes (FC) in expression, which are 2.122 and 1.819, respectively. This explains why the methane production in the group with GAC addition was higher than that in the other groups. In addition, genes such as *fabG* encoding 3-oxoacyl-[acyl-carrier protein] reductase, *aor* encoding ferredoxin oxidoreductase, and *mtsB* encoding coenzyme M methyltransferase corrinoid protein have been upregulated to varying degrees. Illumina sequence reads have been submitted to the SRA NCBI database under BioProjects PRJNA1241488. Figure 4 classifies the differentially expressed genes into five categories: cellular processes, human diseases, genetic information processing, environmental information processing, and metabolism. It can be seen that the largest number of differentially expressed genes are related to the energy metabolism (including methane metabolism) pathway, followed by carbohydrate metabolism. This indicates that GAC can indeed enhance the activity of *M. barkeri* and at the same time increase the expression levels of some genes to accelerate the electron transfer between *M. barkeri* and stainless steel. This is most likely to accelerate this process by increasing the expression of genes related to the methane metabolism pathway, and the gene most closely associated with this is *mtmC*. The KEGG enrichment rates of each gene are shown in Figure S2.

### 3.3. Syntrophy Can Accelerate the Corrosion of Stainless Steel by Methanosarcina barkeri

**Figure 5:**
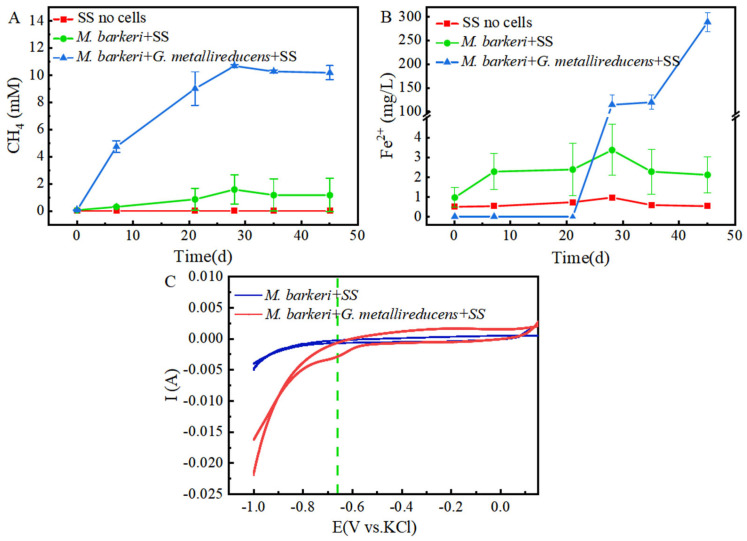
Production of CH_4_ (**A**) and Fe(II) (**B**) changing with time in different experimental groups and the cyclic voltammogram (**C**) during the corrosion of stainless steel by *M. barkeri*.

*M. barkeri* can syntrophically coexist with *G. metallireducens*, accelerating the electron transfer rate and generating methane [24]. In this experiment, stainless steel with no cells was used as the control, and in order to minimize the influence of oxygen anions in nutrients on inhibiting local corrosion [37] and make the experimental groups comparable, stainless steel and 1 mM ethanol or 1 mM sodium acetate were selected as electron donors to construct a co-culture system of *M. barkeri* and stainless steel and a co-culture system of *M. barkeri*, *G. metallireducens*, and stainless steel to explore the enhancement effect of syntrophic metabolism on the corrosion of stainless steel by *M. barkeri*. The changes in the contents of CH_4_ and Fe(II) during the experiment are shown in Figure 5. The co-culture system of *M. barkeri*, *G. metallireducens*, and stainless steel entered the logarithmic growth phase relatively quickly and reached a stable state around the 30th day. During this period, the maximum value of methane production reached 10.67 ± 0.09 mM (Figure 5A), which was higher than the 1.5 mM methane theoretically produced by 1 mM ethanol and 5.13-fold that of the group with *M. barkeri* and stainless steel (*p* < 0.05). Since the main component of the FC medium used for culturing *G. metallireducens* is ferric citrate, a large amount of Fe(II) will be carried during inoculation. In order to eliminate this influence, when measuring the Fe(II) concentration, the part in the inoculum of *G. metallireducens* was deducted, and the curve shown in Figure 5B was obtained. It can also be seen from Figure 5 that the content of Fe(II) in the experimental group of “*M. barkeri* + *G. metallireducens* + SS” was significantly higher than that in the experimental group of “*M. barkeri* + SS” (*p* < 0.05), indicating that it obtained more electrons from stainless steel, which proves that syntrophic symbiotic metabolism can accelerate the corrosion of stainless steel by *M. barkeri*. This is consistent with Nardy’s description that metal corrosion is affected by complex processes such as different electrochemical reactions carried out by different microorganisms [38].

As shown in Figure 5C, the area enclosed by the cyclic voltammogram obtained from the “*M. barkeri* + SS” experimental group is relatively small, indicating that the capacitance of this system is in a relatively small state. In contrast, the area enclosed by the curve obtained from the “*M. barkeri* + *G. metallireducens* + SS” experimental group is significantly larger than that of the previous experimental group (*p* < 0.05), suggesting that more *M. barkeri* adhered to the surface of the electrode, and the capacitance of the system increased accordingly. In addition, there are no obvious oxidation peaks and reduction peaks in the “*M. barkeri* + SS” experimental group. However, in the “*M. barkeri* + *G. metallireducens* + SS” system, a reduction peak appears at around −0.66 V, which is similar to the reduction peak in the methane production of the microbial electrolysis cell experimented on by Yufang Wei [39]. This indicates that at this potential, *M. barkeri* obtained electrons from the stainless steel on the electrode and carried out the reaction of reducing CO_2_ to generate CH_4_. However, the potential corresponding to the reduction peak in the experimental group with the addition of GAC is −0.28 V, which is different from the potential of the reduction peak in the experimental group with the addition of *G. metallireducens*. This may be because GAC and *G. metallireducens* have different overpotentials. At the same time, the different corrosion degrees of the stainless steel between the two groups will also lead to different overpotentials. Meanwhile, Nelabhotla and Dinamarca [40] pointed out that transferring a certain number of electrons at a lower potential is more efficient than transferring the same number of electrons at a higher potential, which may also be the reason why the methane production in the experimental group with the addition of *G. metallireducens* is higher than that in the experimental group with the addition of GAC. In conclusion, the enlarged capacitance and the existence of the reduction peak both prove that in the presence of *G. metallireducens*, *M. barkeri* is more likely to adhere to the stainless steel electrode, transfer electrons with it, and carry out the reaction of reducing CO_2_ to generate CH_4_, thus accelerating the corrosion of the stainless steel.

The corresponding strains of *G. metallireducens* and *M. barkeri* were also captured within the corrosion pits of the stainless steel in the experimental group with the addition of *G. metallireducens* (Figure 3D), and the two strains were attached to each other. Meanwhile, the Fe weight percentage in the experimental group with the addition of *G. metallireducens* was 70.26%, which was lower than that in the “*M. barkeri* + SS” group. This indicates that *G. metallireducens* indeed participated in and accelerated the corrosion of stainless steel by *M. barkeri*. Since the iron content of the corroded stainless steel is similar to that in the experimental group with the addition of GAC, it is speculated that the acceleration rates of the two are similar, but the specific situation still needs to be verified by experiments.

### 3.4. Mechanism Analysis of Syntrophy Accelerating the Corrosion of Stainless Steel by Methanosarcina barkeri

**Figure 6:**
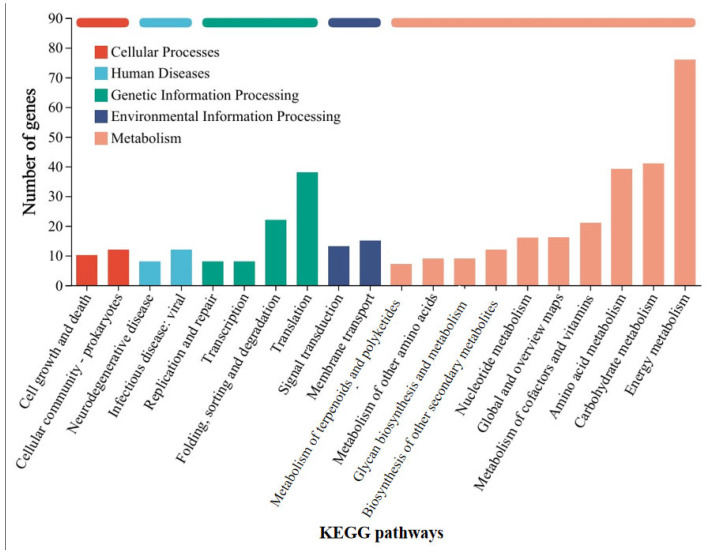
KEGG pathway classification statistics chart of differentially expressed genes between the “*M. barkeri* + *G. metallireducens* + SS” group and the “*M. barkeri* + SS” group.

Similarly, in order to understand the mechanism by which *G. metallireducens* accelerates the corrosion of stainless steel by *M. barkeri*, transcriptome analysis was still carried out on *M. barkeri* after the experiment was completed. Some genes in the “*M. barkeri* + *G. metallireducens* + SS” experimental group whose gene expressions were upregulated were compared with those in the “*M. barkeri* + SS” experimental group, and the difference multiples of gene expression levels greater than 2.5 were selected, and the gene table shown in Table 2 was obtained. The other upregulated genes and specific values are shown in Table S3. As shown in Table 2, among the upregulated genes, apart from some genes encoding hypothetical proteins with relatively large upregulation multiples, *fwdC* encoding furan dehydrogenase and *mtbC* encoding methyltransferase-like proteins, both belonging to the methane metabolic pathway, show relatively large fold differences, which are 12.816 and 2.563, respectively. Furan dehydrogenase was initially regarded as an intermediate in CO-based methanogenesis [41]. By catalyzing the conversion of formate to carbon monoxide, *fwdC* indirectly provides electrons for the methane-generating process. *M. barkeri* utilizes these electrons along with hydrogen or carbon dioxide to produce methane. Therefore, the *fwdC* gene promotes the methane-generating process by enhancing the conversion of formate. It has been newly discovered that CO_2_ can be captured as a formyl group branched on the amine moiety of the methanofuran cofactor, which also represents hydrogenotrophic methanogenesis [42]. However, both *M. barkeri* and *G. metallireducens* use H_2_ as a mediator without producing hydrogen, and 316 L stainless steel abiotically produces little or no H_2_ [43]. Thus, there is almost no hydrogenotrophic methane production in this system. *mtbC* is recognized as a methyltransferase gene that initiates methanogenesis from methylamine [44]. However, the research of S. L. Tsola found that *mtbC* can also participate in the anaerobic conversion of dimethyl sulfide to methane, challenging the view that “substrate-specific methyltransferases are used for methylotrophic methanogenesis” [45]. Therefore, *mtbC* is indeed likely to participate in the process of converting carbon dioxide to methane. On the other hand, after classifying the differentially expressed genes according to KEGG pathways in the same way, Figure 6 was obtained, and the KEGG enrichment rates of each gene are shown in Figure S3. As can be seen from Figure 6, most of the differentially expressed genes belong to the energy metabolism (including methane metabolism) pathway. These indicate that when the syntrophic bacterium *G. metallireducens* is co-cultured with *M. barkeri*, different from the genes affected by GAC, it will lead to the high expression of *mtbC* and *fwdC* to improve the ability of *M. barkeri* to utilize the electrons of stainless steel to complete its own metabolism and produce methane.

## 4. Conclusions

This study demonstrated that GAC and the syntrophy *G. metallireducens* can accelerate the corrosion of stainless steel by *M. barkeri*. Both of them promote the conversion of CO_2_ into CH_4_ through genes encoding methyltransferases. The acceleration in the GAC system mainly increases the expression level of methane metabolism gene *mtmC*, and that in the *G. metallireducens* system mainly increases the expression level of *mtbC* and *fwdC*. This study investigates the effects of the two on the corrosion of *M. barkeri* on stainless steel, filling a gap in this field and providing theoretical guidance for microbial anti-corrosion, which is of practical significance. However, the effects of GAC and *G. metallireducens* dosage on corrosion, as well as the synergistic mechanisms of *mtmC*, *fwdC*, and other functional genes in the complex stainless steel system, remain unclear.

## Figures and Tables

**Table 1:** Selected genes with relatively high differential upregulation of *M. barkeri* genes in the “*M. barkeri* + SS + GAC” experimental group and the “*M. barkeri* + SS” experimental group.

Gene ID	KO Name	Gene Description	Pathway Definition	FC ^a^ (MB-GAC/MB)	Log_2_FC ^b^ (MB-GAC/MB)
MSBRM_3095	------	DUF378 domain-containing protein	------	23.949	4.582
MSBRM_3339	------	hypothetical protein	------	24.252	4.600
MSBRM_2922	------	STARP antigen	------	2.462	1.300
MSBRM_2359	------	hypothetical protein	------	12.126	3.600
MSBRM_1961	------	hypothetical protein	------	14.551	3.863
MSBRM_2245	------	putative membrane-associated protein	------	13.338	3.738
MSBRM_1851	*mtmC*	Monomethylamine methyltransferase corrinoid protein	Methane metabolism	7.276	2.863
MSBRM_2105	*fabG*	3-oxoacyl-[acyl-carrier protein] reductase	Biotin metabolism Fatty acid biosynthesis	3.638	1.863
MSBRM_0533	*cofD*	2-phospho-L-lactate transferase	Methane metabolism	2.122	1.085
MSBRM_1439	*aor*	Tungsten containing aldehyde:ferredoxin oxidoreductase	Pentose phosphate pathway	1.819	0.863
MSBRM_1541	*mtsB*	Methylthiol:coenzyme M methyltransferase corrinoid protein	Sulfur metabolism	1.819	0.863
MSBRM_1197	*mfnE*	delta 1-pyrroline-5-carboxylate synthetase	Methane metabolism	1.819	0.863
MSBRM_1079	*cbiQ*	Transmembrane component CbiQ of energizing module of cobalt ECF transporter	ABC transporters	1.819	0.863

^a^ FC (stress/control): The fold change of this gene between the two samples; ^b^ Log_2_FC (stress/control): the base-2 logarithm of the fold change of this gene between the two samples; the control represents the control group.

**Table 2:** Selected genes with relatively high differential upregulation of *M. barkeri* genes in the “*M. barkeri* + *G. metallireducens* + SS” experimental group compared with the “*M. barkeri* + SS” experimental group.

Gene ID	KO Name	Gene Description	Pathway Definition	FC(MB_GM/MB)	Log_2_FC(MB_GM/MB)
MSBRM_0413	------	hypothetical protein	------	44.909	5.489
MSBRM_1961	------	hypothetical protein	------	35.886	5.165
MSBRM_3095	------	DUF378 domain-containing protein	------	10.894	3.445
MSBRM_0718	------	collagen triple helix repeat domain protein	------	5.554	2.473
MSBRM_2952	------	hypothetical protein	------	19.968	4.320
MSBRM_2105	*fabG*	3-oxoacyl-[acyl-carrier protein] reductase	Biotin metabolism Fatty acid biosynthesis	6.408	2.680
MSBRM_1954	*trpB*	Tryptophan synthase beta chain	Phenylalanine, tyrosine, and tryptophan biosynthesis	2.563	1.358
MSBRM_1237	*puuE*	4-aminobutyrate aminotransferase	Alanine, aspartate, and glutamate metabolism	2.563	1.358
MSBRM_0462	*mtbC*	Dimethylamine methyltransferase corrinoid protein	Methane metabolism	2.563	1.358
MSBRM_0475	*ipdC*	Pyruvate decarboxylase	Tryptophan metabolism	2.563	1.358
MSBRM_0157	*pylC*	Pyrrolysine synthetase	Lysine biosynthesis	2.563	1.358
MSBRM_2496	*------*	8-oxoguanine DNA glycosylase	Base excision repair	2.563	1.358
MSBRM_0641	*fwdC*	Formylmethanofuran dehydrogenase	Methane metabolism	12.816	3.680

## Data Availability

The original sequence data were submitted to the Sequence Read Archive (SRA) NCBI database under BioProjects PRJNA1241488.

## Supplementary Materials

The following supporting information can be downloaded a: https://www.mdpi.com/article/10.3390/microorganisms13061278/s1: Figure S1: Atomic contents near *M. barkeri* in each experimental group; Figure S2: KEGG enrichment analysis (MB-GAC vs. MB); Figure S3: KEGG enrichment analysis (MB-GM vs. MB); Table S1: Contents of each atom near *M. barkeri* (the scanning object is indicated in the brackets); Table S2: Genes with relatively high differential upregulation of *M. barkeri* genes in the “*M. barkeri* + SS + GAC” experimental group and the “*M. barkeri* + SS” experimental group; Table S3: Genes with relatively high differential upregulation of *M. barkeri* genes in the “*M. barkeri* + *G. metallireducens* + SS” experimental group compared with the “*M. barkeri* + SS” experimental group.
