# Supplementary material for: Activated Carbon and Syntrophy Accelerate the Corrosion of Stainless Steel Under Strict Anaerobic Conditions by Methanosarcina barkeri
Source: Microorganisms. 2025 May 30;13(6):1278. doi: 10.3390/microorganisms13061278 (PMC12195604; doi:10.3390/microorganisms13061278)
Supplement: Supplementary file 1 [file microorganisms-13-01278-s001.zip › microorganisms-3641066-supplementary.pdf]

Supplementary Material

1 Supplementary Figures and Tables

1.1 Supplementary Figures

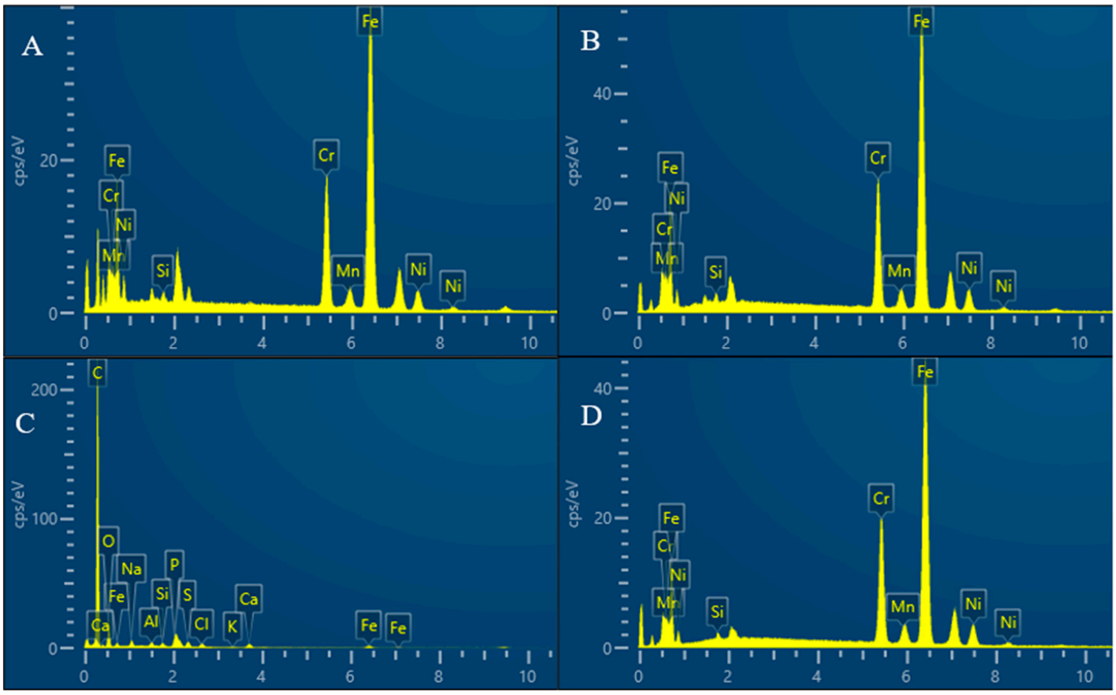

Supplementary Figure S1. Atomic Contents near *M. barkeri* in Each Experimental Group. (**A**: *M. barkeri* + SS (SS); **B**: *M. barkeri* + SS + GAC (SS); **C**: *M. barkeri* + SS + GAC (GAC); **D**: *G. metallireducens* + *M. barkeri* + SS (SS))

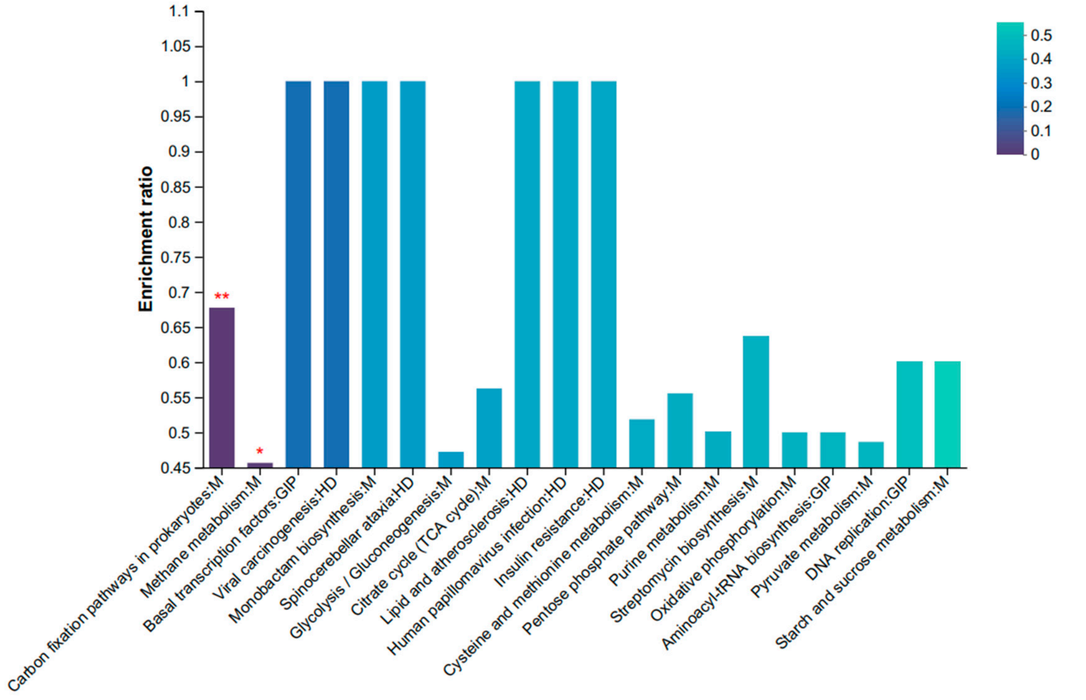

Supplementary Figure S2. KEGG Enrichment Analysis (MB-GAC vs MB)

Note: The horizontal axis represents the name of the pathway, and the vertical axis represents the enrichment rate (The ratio of the number of genes in the gene set annotated to this pathway to the number of genes annotated to this pathway among all genes. The larger the Rich factor, the greater the degree of enrichment). The color represents the significance of enrichment, that is, the P-value. The darker the color, the more significantly enriched the pathway is. Among them, P-value < 0.001 is marked as ***, P-value < 0.01 is marked as **, P-value < 0.05 is marked as *, and the color gradient on the right represents the magnitude of the P-value.

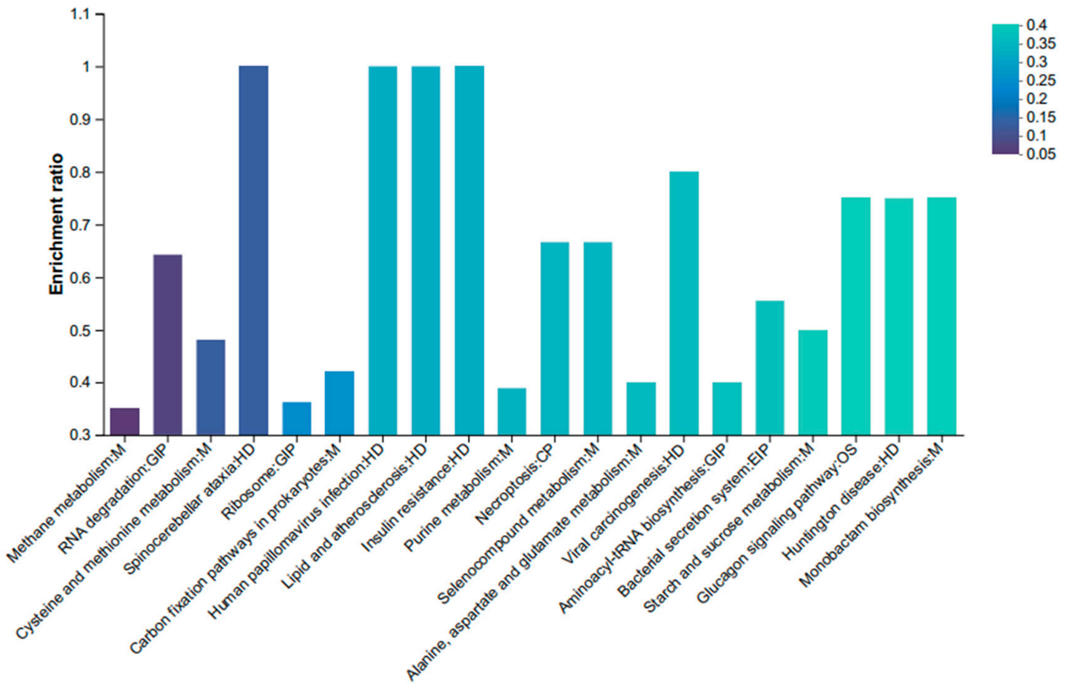

Supplementary Figure S3. KEGG Enrichment Analysis (MB-GM vs MB)

Note: The horizontal axis represents the name of the pathway, and the vertical axis represents the enrichment rate (The ratio of the number of genes in the gene set annotated to this pathway to the number of genes annotated to this pathway among all genes. The larger the Rich factor, the greater the degree of enrichment). The color represents the significance of enrichment, that is, the P-value. The darker the color, the more significantly enriched the pathway is. Among them, P-value < 0.001 is marked as ***, P-value < 0.01 is marked as **, P-value < 0.05 is marked as *, and the color gradient on the right represents the magnitude of the P-value.

1.2 Supplementary Tables

Supplementary Table S1. Contents of Each Atom near *M. barkeri* (The Scanning Object is Indicated in the Brackets)

Wt (%)<br>Group	Si	Cr	Mn	Ni	Fe	C	O
M. barkeri +SS（SS）	1.30	19.92	0.97	7.24	70.41	---	---
M. barkeri +SS+GAC（SS）	1.41	19.76	0.91	7.65	70.27	---	---
M. barkeri +SS+GAC(GAC)	0.05	---	---	---	0.15	87.75	11.44
G. metallireducens+ *M. barkeri* +SS（SS）	1.025	20.05	1.02	7.63	70.26	---	---

Supplementary Table S2. Genes with Relatively High Differential Upregulation of *M. barkeri* Genes in the "*M. barkeri* + SS + GAC" Experimental Group and the "*M. barkeri* + SS" Experimental Group

Gene_id	Gene description	MB	MB_GAC	FC(MB_GAC/MB)	Log2FC(MB_GAC/MB)	Pathway_definition
MSBRM_3095	DUF378 domain-containing protein	6.99	205.28	23.949	4.582	------
MSBRM_3339	hypothetical protein	2.95	89.77	24.252	4.600	------
MSBRM_2922	STARP antigen	23.43	79.55	2.462	1.300	------
MSBRM_2359	hypothetical protein	2.14	33.6	12.126	3.600	------
MSBRM_1961	hypothetical protein	0	33.63	14.551	3.863	------
MSBRM_2245	putative membrane associated protein	0	58.89	13.338	3.738	------
MSBRM_1851	Monomethylamine methyltransferase corrinoid protein	1.48	14.31	7.276	2.863	Methane metabolism
MSBRM_2105	3-oxoacyl-[acyl-carrier protein] reductase	1.33	6.47	3.638	1.863	Biotin metabolism; Fatty acid biosynthesis; Prodigiosin biosynthesis
MSBRM_0533	2-phospho-L-lactate transferase	1.91	5.52	2.122	1.085	Methane metabolism
MSBRM_1439	Tungsten-containing aldehyde: ferredoxin oxidoreductase	1.38	3.5	1.819	0.863	Pentose phosphate pathway
MSBRM_0228	3-isopropylmalate dehydratase small subunit	6.59	13.77	1.617	0.693	C5-Branched dibasic acid metabolism; Valine, leucine and isoleucine biosynthesis
MSBRM_1541	Methylthiol: coenzyme M methyltransferase corrinoid protein	1.1	2.7	1.819	0.863	Sulfur metabolism
MSBRM_1197	delta 1-pyrroline-5-carboxylate synthetase	1.43	3.48	1.819	0.863	Methane metabolism
MSBRM_3096	Deoxyribodipyrimidine photolyase%2C type II	0.63	1.59	1.819	0.863	------
MSBRM_0668	Phosphate transport system regulatory protein PhoU	1.49	3.6	1.819	0.863	------
MSBRM_0812	YbbM seven transmembrane helix protein	1.15	2.84	1.819	0.863	------
MSBRM_2019	Methyltransferase	1.37	3.34	1.819	0.863	------

MSBRM_1079	Transmembrane component CbiQ of energizing module of cobalt ECF transporter	1.18	2.89	1.819	0.863	ABC transporters
MSBRM_2468	hypothetical protein	1.06	2.63	1.819	0.863	------
MSBRM_3406	SSU ribosomal protein S17e	12.38	23.62	1.819	0.863	Ribosome
MSBRM_2444	Formaldehyde activating enzyme	5.77	10.67	1.415	0.500	Methane metabolism
MSBRM_0648	Formylmethanofuran dehydrogenase subunit A	0.95	2.01	1.516	0.600	Methane metabolism
MSBRM_0768	UDP-N-acetylenolpyruvoylglucosamine reductase	1.84	3.82	1.516	0.600	Amino sugar and nucleotide sugar metabolism; Biosynthesis of nucleotide sugars; Peptidoglycan biosynthesis
MSBRM_2823	Queuosine biosynthesis QueD%2C PTPS-I	7.29	13.48	1.516	0.600	Folate biosynthesis
MSBRM_1566	7%2C8-didemethyl-8-hydroxy-5-deazariboflavin synthase subunit 1	2.06	4.24	1.516	0.600	Methane metabolism

Supplementary Table S3. Genes with Relatively High Differential Upregulation of *M. barkeri* Genes in the "*M. barkeri* + *G. metallireducens* + SS" Experimental Group Compared with the "*M. barkeri* + SS" Experimental Group

Gene ID	Gene description	MB	MB_GM	FC(MB_GM/MB)	Log2FC (MB_GM/MB)	Pathway definition
MSBRM_0413	hypothetical protein	0	35.7	44.909	5.489	------
MSBRM_1961	hypothetical protein	0	116.08	35.886	5.165	------
MSBRM_3095	DUF378 domain-containing protein	6.99	130.36	10.894	3.445	------
MSBRM_0718	collagen triple helix repeat domain protein	2.63	27.25	5.554	2.473	------
MSBRM_2952	hypothetical protein	0	115.45	19.968	4.320	------
MSBRM_2105	3-oxoacyl-[acyl-carrier protein] reductase	1.33	15.62	6.408	2.680	Biotin metabolism; Fatty acid biosynthesis; Prodigiosin biosynthesis
MSBRM_1954	Tryptophan synthase beta chain	1.97	9.51	2.563	1.358	Phenylalanine, tyrosine and tryptophan biosynthesis; Glycine, serine and threonine metabolism
MSBRM_2695	AMP/CMP kinase AK6	1.87	12.94	3.845	1.943	Ribosome biogenesis in eukaryotes; Nucleotide metabolism; Purine metabolism
MSBRM_3511	Chemotaxis protein methyltransferase CheR	1.99	9.49	2.563	1.358	Bacterial chemotaxis; Two-component system
MSBRM_1237	4-aminobutyrate aminotransferase	1.19	5.73	2.563	1.358	Alanine, aspartate and glutamate metabolism; Propanoate metabolism; beta-Alanine metabolism; Butanoate metabolism
MSBRM_2596	LSU ribosomal protein L32e	7.68	28.92	2.136	1.095	Ribosome; Coronavirus disease - COVID-19
MSBRM_0462	Dimethylamine methyltransferase corrinoid protein	1.56	7.29	2.563	1.358	Methane metabolism
MSBRM_0927	Dimethylamine methyltransferase corrinoid protein	1.36	6.41	2.563	1.358	Methane metabolism

MSBRM_2186	Excinuclease ABC subunit C	0.53	2.55	2.563	1.358	Nucleotide excision repair
MSBRM_2157	Chaperone protein DnaK	0.5	2.43	2.563	1.358	RNA degradation;Tuberculosis; Longevity regulating pathway - worm
MSBRM_0475	Pyruvate decarboxylase	0.5	2.44	2.563	1.358	Tryptophan metabolism
MSBRM_0157	Pyrrolysine synthetase	0.79	3.79	2.563	1.358	Lysine biosynthesis
MSBRM_2496	8-oxoguanine DNA glycosylase	1.35	6.35	2.563	1.358	Base excision repair
MSBRM_0992	Inorganic pyrophosphatase	2.14	9.8	2.563	1.358	Oxidative phosphorylation
MSBRM_0641	Formylmethanofuran dehydrogenase (molybdenum) subunit C	0.05	1.17	12.816	3.680	Methane metabolism
MSBRM_0413	hypothetical protein	0	35.7	44.909	5.489	------
MSBRM_1961	hypothetical protein	0	116.08	35.886	5.165	------
MSBRM_3095	DUF378 domain-containing protein	6.99	130.36	10.894	3.445	------
MSBRM_0718	collagen triple helix repeat domain protein	2.63	27.25	5.554	2.473	------
MSBRM_2952	hypothetical protein	0	115.45	19.968	4.320	------
MSBRM_2105	3-oxoacyl-[acyl-carrier protein] reductase	1.33	15.62	6.408	2.680	Biotin metabolism; Fatty acid biosynthesis; Prodigiosin biosynthesis
MSBRM_1954	Tryptophan synthase beta chain	1.97	9.51	2.563	1.358	Phenylalanine, tyrosine and tryptophan biosynthesis; Glycine, serine and threonine metabolism
MSBRM_2695	AMP/CMP kinase AK6	1.87	12.94	3.845	1.943	Ribosome biogenesis in eukaryotes; Nucleotide metabolism;Purine metabolism
MSBRM_3511	Chemotaxis protein methyltransferase CheR	1.99	9.49	2.563	1.358	Bacterial chemotaxis;Two-component system
